# ZrTe_2_/CrTe_2_: an epitaxial van der Waals platform for spintronics

**DOI:** 10.1038/s41467-022-30738-1

**Published:** 2022-05-27

**Authors:** Yongxi Ou, Wilson Yanez, Run Xiao, Max Stanley, Supriya Ghosh, Boyang Zheng, Wei Jiang, Yu-Sheng Huang, Timothy Pillsbury, Anthony Richardella, Chaoxing Liu, Tony Low, Vincent H. Crespi, K. Andre Mkhoyan, Nitin Samarth

**Affiliations:** 1 Department of Physics and Materials Research Institute, The Pennsylvania State University, University Park, PA 16802 USA; 2 Department of Chemical Engineering and Materials Science, University of Minnesota, Minneapolis, MN 55455 USA; 3 Department of Electrical & Computer Engineering, University of Minnesota, Minneapolis, MN 55455 USA; 4 School of Physics & Astronomy, University of Minnesota, Minneapolis, MN 55455 USA

**Keywords:** Magnetic properties and materials, Spintronics, Topological matter

## Abstract

The rapid discovery of two-dimensional (2D) van der Waals (vdW) quantum materials has led to heterostructures that integrate diverse quantum functionalities such as topological phases, magnetism, and superconductivity. In this context, the epitaxial synthesis of vdW heterostructures with well-controlled interfaces is an attractive route towards wafer-scale platforms for systematically exploring fundamental properties and fashioning proof-of-concept devices. Here, we use molecular beam epitaxy to synthesize a vdW heterostructure that interfaces two material systems of contemporary interest: a 2D ferromagnet (1T-CrTe_2_) and a topological semimetal (ZrTe_2_). We find that one unit-cell (u.c.) thick 1T-CrTe_2_ grown epitaxially on ZrTe_2_ is a 2D ferromagnet with a clear anomalous Hall effect. In thicker samples (12 u.c. thick CrTe_2_), the anomalous Hall effect has characteristics that may arise from real-space Berry curvature. Finally, in ultrathin CrTe_2_ (3 u.c. thickness), we demonstrate current-driven magnetization switching in a full vdW topological semimetal/2D ferromagnet heterostructure device.

## Abstract (web summary)

Van der Waals heterostructures offer the potential of integrating multiple material layers into a single device to achieve new functionalities. Here, Ou et al combine ZrTe_2_, a topological semimetal, with CrTe_2_, a 2D ferromagnet, in a single heterostructure and demonstrate spin-orbit torque switching of the 2D ferromagnet by current in the topological semimetal.

## Introduction

Van der Waals (vdW) materials are an exciting playground for the discovery of emergent behavior in electrical, optical, and thermal properties in the two-dimensional (2D) limit and are potentially attractive for next-generation device applications^1–8^. The recent demonstration of long-range ferromagnetic order in 2D vdW materials has opened another new avenue to study magnetism in atomically thin films^9–15^. While many studies of 2D vdW ferromagnets have focused on mechanically exfoliated flakes^16–19^, 2D vdW ferromagnets embedded in heterostructures create new opportunities for manipulating and engineering magnetic properties^20–22^. Such multilayer structures can potentially serve as building blocks for 2D magnetic and spintronics applications^14,15^. For example, chiral magnetic textures have been observed in mechanically stacked heterostructures using the vdW ferromagnet Fe_3_GeTe_2_^23,24^. Spin-orbit torque (SOT)-assisted magnetization switching has also been reported in layered vdW ferromagnets interfaced with heavy metals^25–27^.

Amongst the candidate 2D ferromagnets, 1T-CrTe_2_ has an interesting combination of properties. Bulk 1T-CrTe_2_ is a known ferromagnetic material with a Curie temperature, *T*_c_, above room temperature. This persists even in flakes exfoliated down to thicknesses of tens of nanometers^28–31^. Synthesized epitaxial thin films of this material also show a relatively high *T*_c_ down to the quasi-2D regime^32,33^. An in-plane-to-out-of-plane transition of the magnetic easy axis in 1T-CrTe_2_ may be controlled through thickness and strain in thin films^34,35^. Finally, 1T-CrTe_2_ single crystals show reasonable stability against degradation after being exposed to the atmosphere^34^.

Here, we report the synthesis by molecular beam epitaxy (MBE) of full vdW heterostructures that interface ultrathin ferromagnetic 1T-CrTe_2_ films with ZrTe_2_, a candidate topological Dirac semimetal. We note that a recent study^36^ has demonstrated MBE growth of vdW heterostructures that epitaxially combine relatively thick (10 u.c.) CrTe_2_ with a topological insulator (Bi_2_Te_3_) but we are not aware of any published reports of epitaxial vdW 2D ferromagnet/topological semimetal heterostructures. We use in vacuo angle-resolved photoemission spectroscopy (ARPES) to measure the band dispersion of metallic 1T-CrTe_2_ and find that it is consistent with first-principles calculations. Measurements of the anomalous Hall effect (AHE) demonstrate robust ferromagnetism in both single 1T-CrTe_2_ epilayers grown on sapphire and in vdW sapphire/ZrTe_2_/CrTe_2_ heterostructures. In thick films (12 u.c.) of 1T-CrTe_2_ layers grown directly on sapphire or on ZrTe_2_, we observe an AHE whose magnetic field dependence is suggestive of real-space Berry curvature effects. We further use the AHE to demonstrate the persistence of ferromagnetic order in one unit cell of 1T-CrTe_2_ grown epitaxially on ZrTe_2_, thus realizing a wafer-scale spintronics platform that epitaxially interfaces a 2D ferromagnet with a topological semimetal. Finally, we demonstrate current-induced magnetization switching in an ultrathin full vdW ZrTe_2_/CrTe_2_ heterostructure device, where the spin-orbit torque efficiency of the ZrTe_2_ layer is evaluated via spin-torque ferromagnetic resonance (ST-FMR) measurements in ZrTe_2_/Ni_80_Fe_20_ (permalloy, Py) heterostructures.

## Results and discussion

### MBE growth and characterizations of the ZrTe_2_/CrTe_2_ heterostructures

**Fig. 1:**
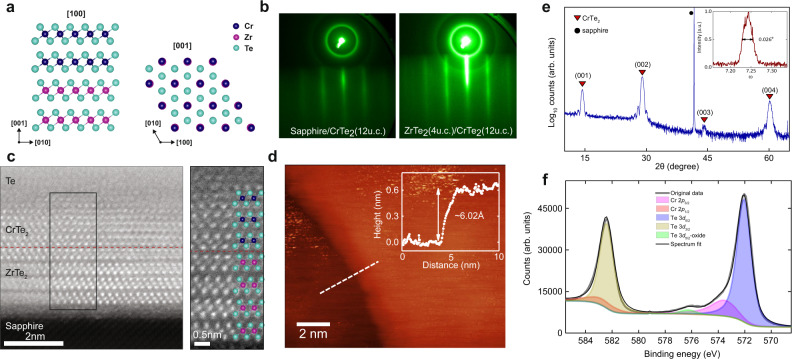
MBE and characterization of 1T-CrTe_2_ thin films. **a** Schematics of the 1 T phase of CrTe_2_ grown on ZrTe_2_. Note that [100], [010], and [001] correspond to $\left[\bar{1}2\bar{1}0\right]$, $\left[2\bar{1}\bar{1}0\right]$, and [0001] in the 4 axis Miller–Bravais notation. **b** RHEED patterns of the single layer of CrTe_2_ (12 u.c.) and ZrTe_2_ (4 u.c.)/CrTe_2_ (12 u.c.) heterostructure. The substrate is sapphire in both cases and the electron beam is directed along the [11$\bar{2}$0] orientation of sapphire. **c** HAADF-STEM images of the ZrTe_2_ (4 u.c.)/CrTe_2_ (3 u.c.) heterostructure viewed in cross-section. The images have been low-pass filtered for clarity. **d** STM image of a ZrTe_2_ (4 u.c.)/CrTe_2_ (1 u.c.) sample. The line scan shows the thickness of the 1 u.c. CrTe_2_. **e** XRD 2$\theta$ scan of a 12 u.c. CrTe_2_ thin film. **f** XPS spectrum of an 18 u.c. thick CrTe_2_ film. The small Te oxide shoulder is due to the absence of a capping layer in this sample. The counts in **e** and **f** are in arbitrary units (arb.units.).

We grew single-layer 1T-CrTe_2_ and ZrTe_2_/CrTe_2_ (Fig. 1a) heterostructures on (001) sapphire substrates by co-deposition from Cr (Zr) and Te sources in an MBE chamber with a base pressure of ~1 × 10^−10^ mbar. The growth was monitored with 13 keV reflection high energy electron diffraction (RHEED). Sharp streaky RHEED patterns (Fig. 1b) indicated the epitaxial growth of the materials (see Materials and Methods for details). The film thickness during growth was controlled by the deposition time (~1 u.c. per 10 min for CrTe_2_ growth, ~1 u.c. per 25 min for ZrTe_2_) as calibrated from x-ray reflectometry. To protect the thin films from oxidation during ex situ characterization, we deposited a capping layer of ~40 nm Te. The 1T-CrTe_2_ crystal structure belongs to the $P\bar{3}{{{{{\rm{m}}}}}}1$ space group (Fig. 1a). We characterized the crystalline structure of the ZrTe_2_/CrTe_2_ heterostructures using aberration-corrected scanning transmission electron microscopy (STEM), as shown in the cross-sectional high-angle annular dark-field (HAADF) images in Fig. 1c, revealing an atomically flat interface between ZrTe_2_ and CrTe_2_. The atomic alignment between the CrTe_2_ layers matches well to that of 1 T phase CrTe_2_ (Fig.1c). We used energy dispersive X-ray (EDX) spectroscopy to determine the relative concentration of Cr and Te in the CrTe_2_ thin films, where it showed Cr/Te=0.53, indicating limited (if any) Cr intercalation (See Supplementary information). The lattice constants of the 1T-CrTe_2_ thin films grown on sapphire, *a* = *b* = 3.93$\pm$0.06 Å and *c* = 6.02$\pm$0.05 Å $\left(\left\langle {90}^{o}\times {90}^{o}\times {120}^{o}\right\rangle \right)$, were further evaluated from the atomic HAADF-STEM images using sapphire as a reference. The measured in-plane lattice constant is ~3% larger than reported in bulk crystals^28^, possibly because our epitaxial layers are strained. Figure 1d shows the topography of 1 u.c. CrTe_2_ grown on ZrTe_2_ measured via in vacuo transfer to a scanning tunneling microscope (STM). The height profile indicates the thickness of ~6.0 Å for the 1 u.c. CrTe_2_ sample, in good agreement with the TEM results.

Figure 1e shows the x-ray diffraction spectrum of a CrTe_2_ thin film with peaks corresponding to the out-of-plane (001) growth direction as well as peaks from the sapphire substrate. The rocking curve (inset of Fig. 1e) of the CrTe_2_ (001) peak gives a full width at half maximum (FWHM) ~0.03 degree, indicating a reasonable crystallinity in the grown films. Additional reciprocal lattice maps were used to characterize the mosaic spread (see Supplementary information). We also used X-ray photoemission spectroscopy (XPS) to determine the sample composition. Figure 1f shows interference between Te 3*d* and Cr 2*p* XPS spectrum. The presence of chromium was also confirmed by the weak Cr 3*s* and Cr 2*s* peaks (not shown here) as well as curve fitting of reference telluride and Cr° spectra acquired under similar conditions. Peak positions obtained were as follows: Te 3*d*_5/2_ at 572.0 eV, Te 3*d*_3/2_ at 582.4 eV, Cr 2*p*_3/2_ at 573.5 eV, and Cr 2*p*_1/2_ at 582.7 eV respectively. The XPS element analysis gives the Te/Cr concentration ratio ~2:1, which is in good agreement with the STEM-EDX analysis.

**Fig. 2:**
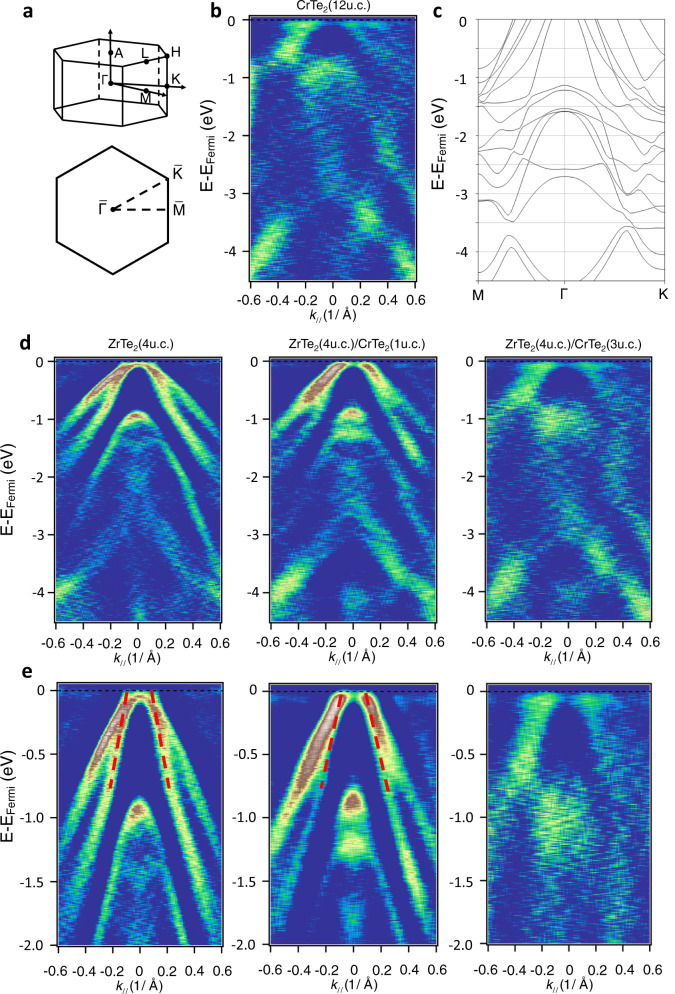
ARPES measurements and DFT calculation of the band structure of 1T-CrTe_2_ thin films. **a** Schematic of the bulk and projected Brillouin zones of CrTe_2_. **b** ARPES spectrum of a single layer of 12 u.c. CrTe_2_ in the $\bar{\Gamma }-\bar{{{{{{\rm{M}}}}}}}$ direction. **c** DFT calculation of the band structure of bulk CrTe_2_. The M and K points are 0.92 and 1.06 Å^−1^ respectively. **d**, **e** ARPES spectrum of the 4 u.c. ZrTe_2_ (left), ZrTe_2_ (4 u.c.)/CrTe_2_ (1 u.c.) (middle), and ZrTe_2_ (4 u.c.)/CrTe_2_ (3 u.c.) (right) with a larger (**d**) and smaller (**e**) binding energy scale. All the ARPES data were taken at 300 K with 21.2 eV excitation from a He lamp. To more clearly highlight the measured band dispersion, we present all plots as second-derivatives with respect to the energy. The red dashed lines are guides to the eyes for the Dirac dispersion in ZrTe_2_.

We measured the band structure of the 1T-CrTe_2_ thin films through in vacuo transfer to an ARPES chamber with excitation from the 21.2 eV ${{{{{\rm{I}}}}}}\alpha$ spectral line of a helium plasma lamp. Photoemitted electrons were detected by a Scienta Omicron DA 30 L analyzer with 6 meV energy resolution. Figure 2a shows the hexagonal Brillouin zone of 1T-CrTe_2_ and Fig. 2b shows the band dispersion of the 1T-CrTe_2_ (001) surface measured along the $\bar{\Gamma }-\bar{{{{{{\rm{M}}}}}}}$ direction at room temperature. The location of the chemical potential within the valence bands indicates the sample is p-type, a fact also confirmed using Hall effect measurements. We performed first-principles density functional theory (DFT) calculations for bulk 1T-CrTe_2_ including spin-orbit coupling with the magnetic moment oriented out-of-plane, obtaining the band structure shown in Fig. 2c. There is good agreement between the ARPES spectrum and the calculated bands (see Supplementary information for more 1T-CrTe_2_ calculations). The asymmetry observed in the ARPES intensity is likely due to matrix element effects^37^.

Figure 2d, e show the ARPES spectra of certain ZrTe_2_/CrTe_2_ heterostructures. The ARPES spectrum of ultrathin 1 u.c. CrTe_2_ grown on ZrTe_2_ largely resembles the band dispersion of ZrTe_2_. We attribute this to the CrTe_2_ 1 u.c. layer (~0.6 nm) being thinner than the mean free path of photoemission electrons near the sample surface, rendering the ARPES signal from the ZrTe_2_ layer beneath still detectable. Note the linear dispersion of the Dirac band from ZrTe_2_, supporting the presence of a topological Dirac semimetal phase of the 4 u.c. ZrTe_2_ film^38,39^. As the CrTe_2_ film becomes thicker (3 u.c. CrTe_2_), its ARPES band dispersion looks more similar to the 12 u.c. CrTe_2_ results. The smooth transition of the band dispersion in the ZrTe_2_/CrTe_2_ heterostructure suggests an excellent epitaxy between the two materials and lays the foundation for the observed robust ferromagnetic order in such bilayers as we discuss below.

### Anomalous Hall resistance measurements

**Fig. 3:**
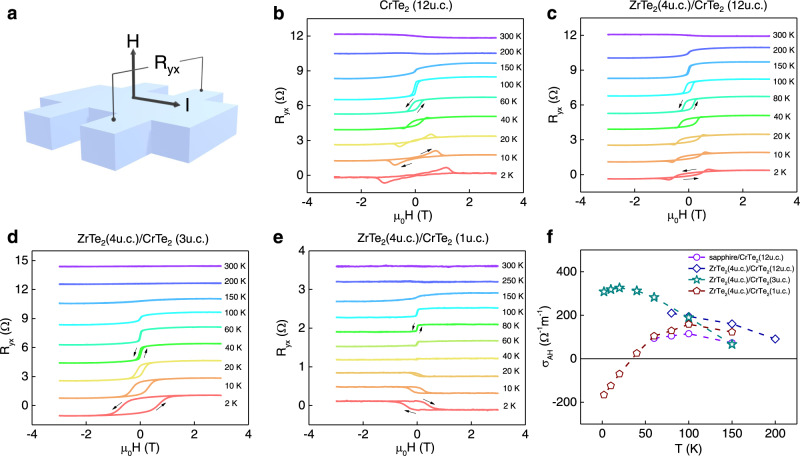
Anomalous Hall resistance measurements on CrTe_2_ layers and ZrTe_2_/CrTe_2_ heterostructure. **a** Schematics of the Hall bar. **b**–**e** Anomalous Hall resistance (AHR) of the 12 u.c. CrTe_2_ (**b**), ZrTe_2_ (4 u.c.)/CrTe_2_ (12 u.c.) (**c**), ZrTe_2_ (4 u.c.)/CrTe_2_ (3 u.c.) (**d**), and ZrTe_2_ (4 u.c.)/CrTe_2_ (1 u.c.) (**e**). The AH resistance data has been offset for clarity. The arrows denote the field sweep directions. **f** Anomalous Hall conductivity of the samples shown from **b** to **e**.

Next, we describe transport measurements of 1T-CrTe_2_ epilayers and ZrTe_2_/CrTe_2_ heterostructures. We measured the Hall resistance of the samples as a function of an out-of-plane magnetic field at various temperatures (details of the longitudinal resistivity measurements are given in the Supplementary information). Figure 3 shows the sample schematics as well as the Hall resistance of the single layer and heterostructures (see Methods for measuring and analyzing the Hall resistance data). Starting with the results in a 12 u.c. (~7.2 nm thick) CrTe_2_ film grown on sapphire, the Hall effect at room temperature shows a small nonlinear Hall signal at low magnetic fields, suggesting weak ferromagnetic order with a Curie temperature (*T*_c_) in the vicinity of room temperature. This is consistent with an earlier report that a 10 nm thick exfoliated flake of 1T-CrTe_2_ has a *T*_c_ above room temperature^29^. Note that earlier reports indicate that *T*_c_ may be enhanced in thin films of CrTe_2_ and other CrTe compounds in CVD-grown samples as compared to thicker films^34,40^.

As we cool the 12 u.c. CrTe_2_ thin film below 200 K, a stronger AH resistance appears with a hysteresis loop whose coercivity continually increases as the temperature falls below 100 K. The hysteretic AHE loop indicates that the 1T-CrTe_2_ thin film exhibits an out-of-plane magnetic easy axis. As we discuss later, we observed an out-of-plane magnetic easy axis in all our CrTe_2_ thin films down to the 1 u.c. limit. This contrasts with the in-plane easy axis observed in bulk exfoliated CrTe_2_ flakes^29–31^ but agrees with other thin-film results^32,34,36^. To understand the magnetic anisotropy behavior in our samples, we used DFT to compute the magnetic anisotropy energy of 1T-CrTe_2_ thin films with different lattice constants (see Supplementary information). We find that the easy axis is sensitive to both lattice strain, in agreement with a previous prediction^35^, and Fermi-level position, making it possible to have different easy axis directions in different experimental settings.

As the sample temperature further decreases below around 40 K, the hysteresis loop of the AHE is replaced by an unconventional shape that has a non-monotonic dependence on the magnetic field, vanishing at both low and high fields and with a peak at an intermediate field. This feature becomes more prominent at low temperatures (2 K). This unusual feature in the Hall resistance has been reported in other ferromagnetic systems where the ferromagnetic order can be of intrinsic origin^41–43^ from ferromagnetic doping^44,45^ or from an interfacial proximity effect^46^. It is regarded as a sign of the topological Hall effect (THE), arising from complex real-space chiral domain structures such as skyrmions^47,48^. This unconventional Hall effect is also seen in CrTe_2_ films of the same thickness grown on ZrTe_2_ (Fig. 3c). We note that a recent study of the AHE in CrTe_2_/Bi_2_Te_3_ has also shown similar magnetic field dependence and has been interpreted in terms of a THE arising from the presence of a non-collinear inversion-symmetry-breaking Dzyaloshinskii–Moriya (DM) interaction due to the interplay between the strong spin-orbit coupling at the CrTe_2_/Bi_2_Te_3_ interface^36^. This interpretation was supported by theoretical simulations. Our observation of a THE-like signature in CrTe_2_ films grown directly on sapphire (a material that does not have strong spin-orbit coupling) suggests that the underlying physics is probably more complex. We might speculate that the interface between CrTe_2_ and sapphire induces a DM interaction but we do not currently have any microscopic model to justify this speculation. The magnitude of the THE in our CrTe_2_/sapphire films is a factor of 6 smaller than that reported in CrTe_2_/Bi_2_Te_3_. Notably, exfoliated CrTe_2_ flakes have been shown to exhibit Néel-type domain walls due to the sixfold crystalline symmetry^31^. Since our CrTe_2_ thin films exhibit an out-of-plane magnetic easy axis, we posit that these Néel-type domain walls transform into a chiral magnetic texture that is an inherent property of CrTe_2_ itself, producing the observed behavior in the Hall measurement. At this stage, we cannot definitively rule out alternative scenarios such as competing ferromagnetic phases that produce AHE of opposite sign^49,50^. However, a detailed analysis of the variation of the Hall effect as a function of temperature and magnetic field suggests that such a trivial alternative scenario is unlikely (see Supplementary information for more discussion). Direct real-space experimental evidence of the chiral domain structures, such as low-temperature magnetic force microscopy or Lorentz transmission electron microscopy, will be needed to definitively determine the nature of the magnetic ordering and its impact on the Hall effect.

We now discuss the ferromagnetism in thinner CrTe_2_ films, focusing on sapphire/ZrTe_2_/CrTe_2_/Te heterostructures that are of higher structural quality than ultrathin CrTe_2_ films grown directly on sapphire. Our measurements show that 3 u.c. thick CrTe_2_ films only begin to show an AHE below 150 K (Fig. 3d). Measurements down to 2 K show a conventional AHE whose magnitude increases monotonically with decreasing temperature; we do not observe any unconventional signatures in the AHE unlike in 12 u.c. thick CrTe_2_ films. We note that a thickness-dependent THE has been observed in other material systems^42,43^, including another CrTe compound epitaxially grown on SrTiO_3_(111) substrates^51^. In our CrTe_2_ films, we tentatively attribute the observed thickness dependence of the Hall effect to the enhancement in magnetic anisotropy which prefers an out-of-plane easy axis that arises from a weakening of the Coulomb screening effect in the 2D limit^34^. We speculate that the 3 u.c. CrTe_2_ film in this case may have different anisotropy energy compared to the thicker sample, such that it no longer satisfies the anisotropy requirement to form a proper phase to exhibit chiral magnetic texture.

To test the robustness of the ferromagnetic order in the true 2D limit of 1T-CrTe_2_, we also measured a heterostructure consisting of only 1 u.c. of CrTe_2_ grown on ZrTe_2_ (Fig. 3e). Like the 3 u.c. CrTe_2_ film, an AHE signal appears at around 150 K. The observation of the AHE unambiguously demonstrates the existence of long-range ferromagnetism even down to the 1 u.c. limit of CrTe_2_ in the heterostructure, confirming vdW 1T-CrTe_2_ as a 2D ferromagnet. In contrast to the thicker films, the magnitude of the AHE shows a non-monotonic dependence on temperature. As the temperature is lowered from 150 K, the magnitude initially increases, reaching a maximum at around 100 K. It then decreases with further lowering of temperature, vanishing at around 40 K and then increasing again but with the opposite sign. This sign reversal of the AHE may be a consequence of the variation of the Berry curvature^52^ induced by the charge transfer between ZrTe_2_ and CrTe_2_. A similar phenomenon has been reported previously in magnetic topological insulator heterostructures^53^. Figure 3f summarizes the measured temperature-dependent anomalous Hall conductivity of the various CrTe_2_ samples (see Supplementary information for more results on the longitudinal magnetoresistance and magnetometry measurements). The difference in the strength of the AHE between sapphire/CrTe_2_ and ZrTe_2_/CrTe_2_ samples might be due to the various interfacial contributions to the AHE and THE, an effect that has been known in other ferromagnetic heterostructures^54^. Without a more detailed microscopic model of the interfacial band structure and its Berry curvature contributions to spin transport, it is not possible for us to provide a more definitive explanation of the variation of the AHE and THE in these different interfacial configurations. We also caution that current shunting through the ZrTe_2_ layer adds complications to a direct comparison of the magnitude of the THE in these different heterostructures.

### Current-induced spin-torque and magnetization switching

**Fig. 4:**
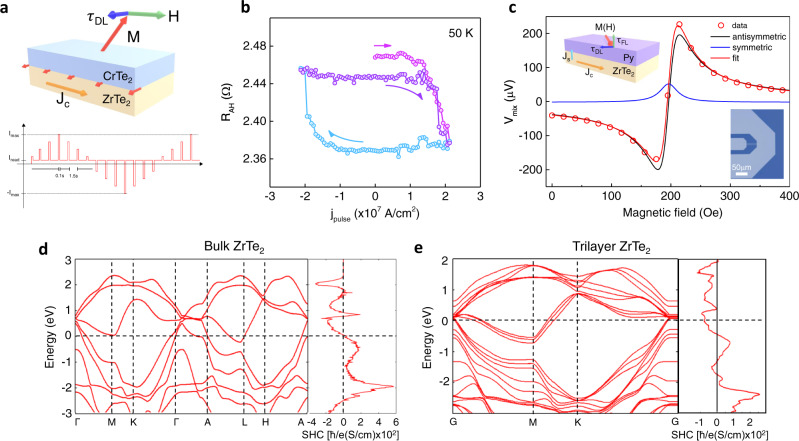
Pulsed current-induced magnetization switching of an ultrathin ZrTe_2_/CrTe_2_ heterostructure device and SOT characterizations of ZrTe_2_. **a** Schematics of the SOT assisted magnetization switching in the ZrTe_2_/CrTe_2_ heterostructure and the writing and reading pulse current sequence. **b** Pulse current-induced magnetization switching of a ZrTe_2_ (8 u.c.)/CrTe_2_ (3 u.c.) at 50 K under an external magnetic field (700 Oe) applied in-plane parallel to the current direction. **c** ST-FMR spectrum of a ZrTe_2_/Py bilayer heterostructure at room temperature. Inset: Optical microscope image and schematic of the ST-FMR device. **d**, **e** DFT calculated band structure and spin Hall conductivity of ZrTe_2_ in the bulk and thin film form, respectively.

Finally, we examine a proof-of-concept spintronic device demonstration of our wafer-scale vdW Dirac semimetal/2D ferromagnet. By using optical lithography, we fabricated 5 μm × 10 μm scale Hall bar devices of a ZrTe_2_(8 u.c.)/CrTe_2_(3 u.c.) heterostructure and used the AHE to probe the response of the CrTe_2_ magnetization to the current flowing in the heterostructure (Fig. 4a). We note that the ZrTe_2_ and CrTe_2_ films have similar conductivities so that current flows in parallel in the two layers. Figure 4b show magnetization switching at 50 K under a pulsed longitudinal current with an external field 700 Oe parallel to the current direction. An out-of-plane magnetic field was first applied to align the magnetization of CrTe_2_ to an initial state before using a pulsed current to switch the magnetization (see Methods). As shown in Fig. 4b, positive and negative current pulses switch the magnetization between two states; the switching edge appears to be step-like, indicating the switching likely involves multi-domain nucleation and expansion under the current-induced spin-orbit torque (SOT) from ZrTe_2_. The average current density for the onset of the magnetization switching of this ZrTe_2_(8 u.c.)/CrTe_2_(3 u.c.) device is about 1.8 × 10^7^ A/cm^2^, which is comparable to the current density needed to switch the magnetization of 3D ferromagnets (e.g., Co, CoFeB) using heavy metals^55,56^. However, we emphasize that because of the complicated nature of the domain nucleation and domain wall motion during the magnetization switching process in micron-meter size devices, it is difficult to directly evaluate the SOT from the switching current density alone^27,56^. We note that we have observed current-switching SOT switching over the temperature range 10 K < T < 90 K without much variation in the threshold switching current density (see Supplementary information for results at additional temperatures and bias fields). Technical constraints prevent us from directly measuring the SOT efficiency using techniques such as ST-FMR and spin pumping at the low temperature required by the Curie temperature of the 3 u.c. CrTe_2_. For a better understanding of the efficiency of ZrTe_2_ as a SOT material, we instead carried out ST-FMR measurements at room temperature on a ZrTe_2_/permalloy (Py) heterostructure. Figure 4c shows the ST-FMR data measured at room temperature on a 50 μm × 10 μm ZrTe_2_(5 nm)/Py(4 nm) device (see Methods). This mixing voltage (V_mix_) signal is the result of the dynamics of the magnetization of the Py layer driven by the SOT induced in the ZrTe_2_ layer by the input radio-frequency (RF) current. The resonance shape of V_mix_ can be separated into a symmetric (S) and antisymmetric (A) Lorentzian component respectively, where the symmetric (antisymmetric) component is proportional to the current-induced SOT (Oersted field). The SOT efficiency, ${\xi }_{{{{{{{\mathrm{FMR}}}}}}}}$, defined as the ratio of the spin current (J_s_) to the charge current (J_c_), is evaluated from the ratio of the symmetric and antisymmetric components^57^: ${\xi }_{{{{{{{\mathrm{FMR}}}}}}}}$ = $\frac{2e}{\hbar }\frac{{J}_{s}}{{J}_{c}}=\frac{S}{A}\frac{e{\mu }_{0}{M}_{s}{t}_{{{ZrTe}}_{2}}{t}_{{Py}}}{\hbar }{\left[1+\left(\frac{{M}_{{{{{{{\mathrm{eff}}}}}}}}}{{H}_{{{{{{{\mathrm{Res}}}}}}}}}\right)\right]}^{1/2}$, where *e* is the charge of the electron, $\hbar$ is the reduced Planck constant, ${\mu }_{0}$ is the permeability of free space, M_S_ is the saturation magnetization of Py, t_ZrTe2(Py)_ is the thickness of the ZrTe_2_(Py) layer, M_eff_ is the effective magnetization and H_Res_ is the resonance field respectively. From the data in Fig. 4c, we obtain ${\xi }_{{{{{{{\mathrm{FMR}}}}}}}}$ = 0.014$\pm$0.005 for ZrTe_2_/Py. With the measured electrical conductivity of ZrTe_2_, ${\sigma }_{{xx}}^{{{{{{{{\mathrm{ZrTe}}}}}}}}_{2}}=3.16\times {10}^{5}{{\mathrm{S{m}}}^{-1}}$, the effective spin Hall conductivity (SHC) of such a ZrTe_2_ thin film is estimated as: ${\sigma }_{{{{{{{\mathrm{SH}}}}}}},{{{{{{\mathrm{effective}}}}}}}}^{{{{{{{{\mathrm{ZrTe}}}}}}}}_{2}}=\left(\hbar /2e\right){\sigma }_{{xx}}^{{{{{{{{\mathrm{ZrTe}}}}}}}}_{2}}{\xi }_{{{{{{{\mathrm{FMR}}}}}}}}\approx \left(\hbar /e\right)2.2\times {10}^{3} {{\mathrm{S{m}}}^{-1}}$. While the ST-FMR result clearly demonstrates charge-to-spin conversion in the Dirac semimetal ZrTe_2_ layer and shows that the spin current generated in the ZrTe_2_ layer is playing an important role in the current-induced magnetization switching experiment in ZrTe_2_/CrTe_2_ heterostructures, the SOT efficiency deduced in this manner is usually only a lower bound of the full SOT efficiency generated inside the SOT material due to the non-ideal interface and interfacial spin transparency. Since the interface in ZrTe_2_/CrTe_2_ is a more coherent one compared to that in ZrTe_2_/Py, we expect a more efficient spin current transfer in the former because of the epitaxial interface and the smooth transition of the band structure as indicated by our ARPES measurements (Fig. 2).

To obtain further insights into the spin-charge conversion generated by ZrTe_2_, we also carried out first-principles calculations of the SHC of both bulk and multilayer ZrTe_2_ (Fig. 4d, e). For the bulk phase, the SHC near the Fermi level is almost zero, and increases in magnitude with electron or hole doping, which is consistent with our relatively small effective SHC of ZrTe_2_ determined via ST-FMR (see above). On the other hand, for the trilayer case, the Fermi level shifts to higher energy with a noticeable broad SHC peak (Fig. 4e). The origin of this SHC peak can be attributed to the presence of topological Dirac nodes in the vicinity of the Fermi level. We have also calculated the SHC for monolayer and bilayer ZrTe_2_. The bilayer shows similar behavior as a trilayer while the monolayer shows quite different behavior (See Supplementary information). Note that the actual Fermi level of ZrTe_2_ may be influenced by the presence of the Py layer, due to charge transfer that results from a difference in their work functions. Nevertheless, due to the broad SHC peak in trilayer ZrTe_2_, we anticipate the Fermi level to be within this conductivity peak.

In conclusion, we used MBE to synthesize 1T-CrTe_2_ thin films and full vdW wafer-scale 1T-CrTe_2_/ZrTe_2_ heterostructures. The out-of-plane magnetic easy axis of these MBE-grown ferromagnetic CrTe_2_ films is of particular interest for studying proof-of-concept spintronics applications such as perpendicular magnetic tunnel junctions and will be technologically relevant once a robust room temperature ferromagnetic state is realized. We observed behavior consistent with a THE in both CrTe_2_ epilayers and heterostructures as indicated by AHE measurements, suggesting CrTe_2_ as a promising material platform for studies of chiral magnetic domain structures. We also demonstrated that long-range ferromagnetic order persists in the heterostructure ZrTe_2_/CrTe_2_ down to the 1 u.c. limit of 1T-CrTe_2_, and we further demonstrated current-induced magnetization switching in an ultrathin ZrTe_2_/CrTe_2_ full vdW heterostructure and characterized the SOT from ZrTe_2_ via ST-FMR measurements. The wafer-scale epitaxial synthesis of heterostructures that cleanly interface the vdW 2D ferromagnet CrTe_2_ with the topological semimetal ZrTe_2_ may provide new opportunities in studying the coexistence of the 2D ferromagnetic and topological phases, interfacial interactions such as proximity induced magnetism in vdW topological semimetals, as well as the SOC interaction and spin-torque phenomena in the true 2D limit.

## Methods

### Sample growth

We deposited single layer 1T-CrTe_2_ thin films and ZrTe_2_/1T-CrTe_2_ thin film heterostructures using MBE in a Scienta Omicron EVO50 system under ultrahigh vacuum (~10^−10^ mB). The sapphire (0001) substrates were outgassed at 600 °C in situ for 1 h to clean the surface before the deposition of the thin films. The epitaxial 1T-CrTe_2_ was grown at a substrate temperature of 280 °C via co-evaporation of Te (purity: 99.99%, Alfa Aesar) and Cr (purity: 99.997%, Alfa Aesar) respectively, with the flux ratio ~40:1. ZrTe_2_ was grown at a substrate temperature of 420 °C. Te was sublimated at a significant overpressure compared to the Zr (purity: grade 702, Kurt J.Lesker) which was evaporated via e-beam at a deposition rate of roughly 0.3${\text{\AA}}$/min. During the growth of ZrTe_2_, the film was annealed periodically throughout the growth under a constant tellurium flux in order to avoid vacancies and mitigate defects. The outgassing and growth temperatures were measured by an infrared camera with an emissivity of 0.7. RHEED was monitored using a 13 keV electron gun during the growth of the samples. Before ex situ characterization and measurements, we capped the samples with 40 nm Te to avoid degradation.

### STM and ARPES measurements

In situ topography was measured at 300 K after transferring MBE-grown samples in vacuo to a Scienta Omicron LT NANOPROBE STM system. We also carried out ARPES measurements at 300 K after in vacuo transfer following the MBE growth of the samples. As excitation, we used the 21.2 eV spectral line from a helium plasma lamp and the emitted photoemission electrons were detected by a Scienta Omicron DA 30 L analyzer with an energy resolution of 6 meV.

### STEM characterization and analysis

Cross-section samples for the STEM study were prepared on an FEI Helios Nanolab G4 dual-beam Focused Ion Beam (FIB) system with 30 keV Ga ions followed by ion-milling at 2 keV to removed damaged surface layers. Amorphous C and Pt were first deposited on the films to protect the surface from damage on exposure to the ion beam. STEM experiments were performed on an aberration-corrected FEI Titan G2 60–300 (S)TEM microscope, which is equipped with a CEOS DCOR probe corrector, monochromator, and a super-X energy dispersive X-ray (EDX) spectrometer. The microscope was operated at 200 and 300 keV with a probe current of 80 pA. HAADF-STEM images were acquired with the probe convergence angle of 25.5 mrad and the detector inner and outer collection angles of 55 and 200 mrad respectively. EDX elemental maps were acquired and analyzed using Bruker Esprit software. The lattice constants were obtained by using the Fourier transform atomic-resolution HAADF-STEM images.

### XRD and XPS characterization

We carried out XRD measurements on X’Pert³ MRD operating in the reflection mode with Cu-Kα radiation (45 kV, 40 mA) and diffracted beam monochromator, using a step scan mode with the step of 0.025°(2θ) and 0.88 s per step. The XPS experiments were performed using a Physical Electronics VersaProbe II instrument equipped with a monochromatic Al kα x-ray source (hν = 1,486.6 eV) and a concentric hemispherical analyzer. Peaks were charge referenced to the CH_x_ band in the carbon 1 s spectra at 284.8 eV. Measurements were made at a takeoff angle of 45° with respect to the sample surface plane. A model line shape for the Te 3*d* spectrum was determined from an exfoliated, oxygen-free Bi_2_Te_3_ sample^58^. We assumed that the shapes would be very similar. The Cr 2*p* line shape was modeled using reference Cr° spectra from the instrument vendor. Three sets of highly constrained doublets (1 each for Cr, Te°, and TeO_x_) were used for the Cr 2*p*-Te 3*d* region. Quantification was done using instrumental relative sensitivity factors (RSFs) that account for the X-ray cross-section and inelastic mean free path of the electrons. The analysis region was ~200 µm in diameter. The sapphire/CrTe_2_ sample without capping layers as measured by XPS was transferred after removal from the MBE chamber into the XPS instrument within 5 min to minimize oxidation.

### Electrical transport characterization

We performed electrical transport measurements in a Quantum Design physical properties measurement system (PPMS) in a Hall bar configuration. Hall bars with lateral dimensions of 1 mm × 0.5 mm were mechanically defined. The Hall resistance of the devices, ${R}_{{yx}}^{* }$, was measured as a function of magnetic field up to 3 T in the temperature range between 2 and 300 K. To determine the anomalous Hall response at a given temperature, we first antisymmetrized the magnetic field dependence of the Hall resistance to remove the longitudinal resistance contribution; then, we subtracted the ordinary Hall resistance${R}_{{H}_{0}}$:${R}_{{yx}}=\frac{\left({R}_{{yx}}^{* }\left(H,T\right)\;-\;{R}_{{yx}}^{* }\left(-H,T\right)\right)}{2}-{R}_{{H}_{0}}$. For samples that do not exhibit any THE, the AH resistance ${R}_{{{{{{{\mathrm{AHE}}}}}}}}$ is then equal to${R}_{{yx}}$. For samples that show a THE signal, the total transverse resistance can be written as${R}_{{yx}}={R}_{{{{{{{\mathrm{AHE}}}}}}}}+{R}_{{{{{{{\mathrm{THE}}}}}}}}$. The anomalous Hall conductivities ${\sigma }_{{{{{{{\mathrm{AH}}}}}}}}$ are calculated as ${\sigma }_{{{{{{{\mathrm{AH}}}}}}}}=\frac{{\rho }_{{{{{{{\mathrm{AHE}}}}}}}}}{\left({\rho }_{{{{{{{\mathrm{AHE}}}}}}}}^{2}+{\rho }_{{xx}}^{2}\left(H=0\right)\right)}$ .

### Pulsed switching measurement

We carried out pulsed current-induced magnetization switching experiments in a PPMS using an external Keithley 2450 source meter and a Keithley 2182 A nanovoltmeter. Before each switching attempt, the magnetization of the device was set and saturated in an initial state by applying a perpendicular magnetic field. In the pulse switching measurement, a train of pulses consisting of a 100 ms current pulse of varying magnitude followed by a 1500 ms pulse of 100 µA was applied under a magnetic field parallel to the current direction, during which we measured the anomalous Hall resistance of our system.

### Spin-torque ferromagnetic resonance measurement

To further study the charge-to-spin conversion in the Dirac semimetal ZrTe_2_ layer, we have performed ST-FMR in a ZrTe_2_/permalloy heterostructure. Without breaking the vacuum, we synthesized ZrTe_2_ (5 nm)/Py (4 nm)/Al (4 nm) heterostructures. These heterostructures were then patterned into 50 um × 10 um bars using standard lithography techniques including a two-step plasma etching process using BCl_3_ and Ar as precursor gases. ST-FMR measurements were performed in these devices using a probe station equipped with a GMW 5201 projected field electromagnet, a Keysight E8257D analog signal generator and a Keithley 2182 A nanovoltmeter. The spectrum was measured using a radiofrequency current ranging from 4 GHz to 6 GHz with an applied in-plane magnetic field up to 1.6 KOe.

### DFT first-principles calculation

Spin-orbit-coupled (SOC) DFT calculations were implemented in the Vienna Ab-initio Simulation Package (VASP)^59–61^ and Quantum Espresso^62^. The lattice constant for 1T-phase CrTe_2_ and ZrTe_2_ was obtained from the experimental result. The z-axis cell dimension is 15 Å for monolayer CrTe_2_ to isolate a layer from its periodic images. The exchange-correlation is treated under GGA PBE approximation^63^ with PAW method^64^. The energy cutoff in all calculations was 500 eV, and the k-point sampling was set as 16 × 16 × 1 and 16 × 16 × 10 centered at Γ for monolayer and bulk structures. The residual force after relaxation is smaller than 0.01 eV/A for all atoms. DFT+U method^65,66^ is used in the calculation and the effective U is 2 eV to make results comparable to previous works^67,68^. Spin Hall conductivity is calculated based on kubo formula using the fitted Hamiltonian, as implemented in the Wannier90 package^69^.

## Supplementary information

Supplementary Information

Peer Review File

## Data Availability

All data for the figures and other Supplementary information that support this work are available upon reasonable request to the corresponding author.
