# Supplementary material for: ZrTe2/CrTe2: an epitaxial van der Waals platform for spintronics
Source: Nat Commun. 2022 May 27;13:2972. doi: 10.1038/s41467-022-30738-1 (PMC9142486; doi:10.1038/s41467-022-30738-1)
Supplement: Supplementary file 1 — Supplementary Information [file 41467_2022_30738_MOESM1_ESM.pdf]

Supplemental Information

ZrTe2/CrTe2: an epitaxial van der Waals platform for spintronics

Yongxi Ou, Wilson Yanez, Run Xiao, Max Stanley, Supriya Ghosh, Boyang Zheng, Wei Jiang, Yu-Sheng Huang, Timothy Pillsbury, Anthony Richardella, Chaoxing Liu, Tony Low, Vincent H. Crespi, K. Andre Mkhoyan, Nitin Samarth

S1 Characterization of the MBE-grown topological semimetal $ZrTe_2$

MBE-grown pristine $ZrTe_2$ has been reported to exhibit massless Dirac fermions in its band dispersion with a Dirac cone at the gamma point, making it an interesting candidate for VdW Dirac semimetals [38,39]. We have grown $ZrTe_2$ on sapphire substrates by following the process described in the Methods sections. Here we provide more details about characterization of our MBE-grown $ZrTe_2$ films. Figure S1A shows the XRD spectrum of a sapphire/$ZrTe_2$ (6u.c.) sample indicating the peaks of $ZrTe_2$ along the out-of-plane (001) growth direction, in good agreement with the literature [39]. Figure S1B (same as Fig. 2 in the main text) shows ARPES measurements of a 4 u.c. thick $ZrTe_2$ film. The parabolic valence band and the linear Dirac dispersion are visible in Fig. S1B. We note that the chemical potential lies below the Dirac point in this sample. This is in good agreement with the results in Ref.[39]. However, this makes direct visualization of the Dirac point difficult. A shift in the Fermi level closer to the Dirac point may be achieved by growing the sample on another type of substrate (e.g. on InAs)[38] and/or via post-growth annealing treatment makes the sample n-doped due to the introduction of Te vacancies. We adopted both methods by annealing a $ZrTe_2$ sample grown on an epi-graphene substrate at 600 °C for 2 hours. As shown in Fig. S1C, the sample indeed became more n-type doped after the annealing treatment resulting in

a clearer Dirac point indicated by the converged linear dispersion. More details about the $ZrTe_2$ synthesis and characterization will be provided in future work. As a control measurement, we also measured the Hall effect in a sapphire/$ZrTe_2$ (4u.c.) sample at various temperatures (Fig. S1D). The lack of any AHE signal in $ZrTe_2$ confirms that the ferromagnetic response in the $ZrTe_2$/$CrTe_2$ samples described in the main text originates in the 1T-$CrTe_2$ layer.

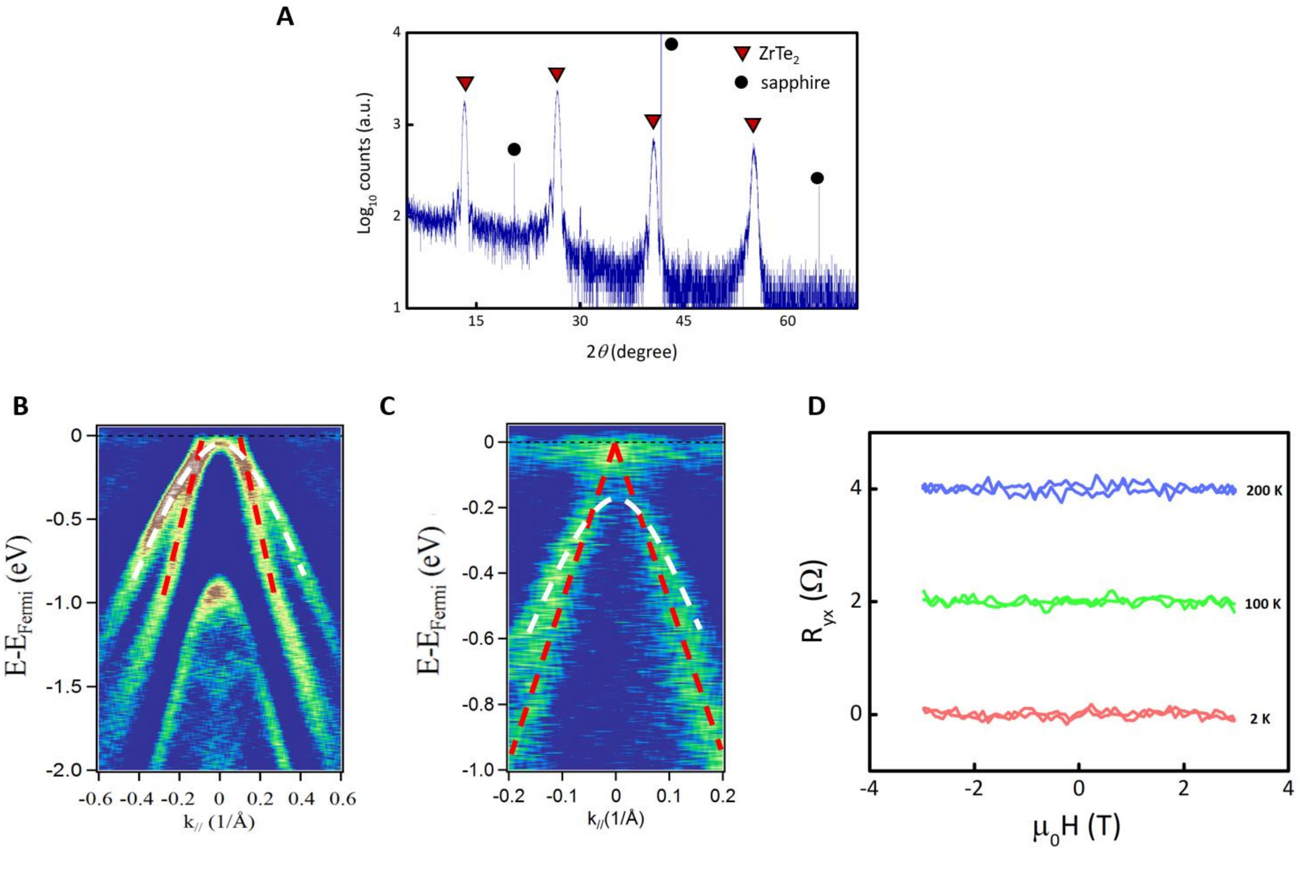

Figure S1. (A) XRD 2θ scan of a sapphire/$ZrTe_2$(6u.c.) sample. (B and C) ARPES spectrum on (B) a sapphire/$ZrTe_2$(4u.c.) sample and (C) an epi-graphene/$ZrTe_2$(10u.c.) sample at room temperature (light source: 21.2 eV). Both ARPES plots are second-derivatives. The red and white

dashed lines are guidance to the eyes. (D) Anomalous Hall resistance of a sapphire/$ZrTe_2$(4u.c.) thin film at various temperature. The AH resistance data has been offset for clarity.

S2 STEM imaging and energy dispersive X-ray (EDX) spectroscopy of the 1T-$CrTe_2$

Figure S2A shows the HAADF-STEM image of a 1T-$CrTe_2$ thin film. The elemental distribution and composition in these MBE-grown $CrTe_2$ thin films were evaluated via the STEM-EDX mapping (Fig. S2B). The EDX line-scan in Fig. 2C shows that Cr atoms are clearly confined to the $CrTe_2$ layer and no oxygen signal is seen in the material layer, indicating it is pristine. From this EDX mapping analysis, we determined the Cr to Te ratio in the grown $CrTe_2$ thin film to be 0.53, which is very close to 1:2, indicating little (if any) Cr intercalation into the films (Fig. S2C).

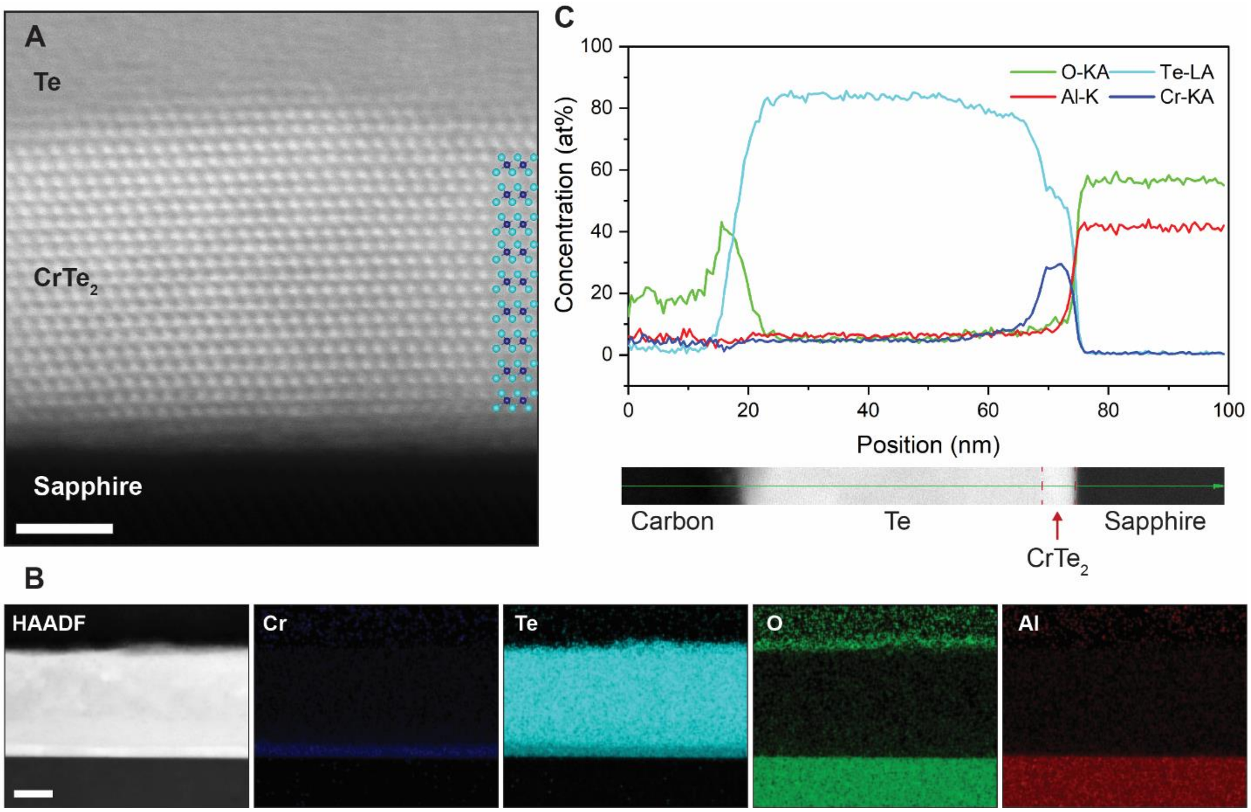

Figure S2. (A) HAADF-STEM images of the sapphire/$CrTe_2$(12u.c.) sample. The image has been low-pass filtered for clarity. Scale bar is 2 nm. (B) A set of STEM-EDX elemental maps of a

sapphire/$CrTe_2$(12u.c.)/Te sample shown along with the HAADF-STEM image acquired with the maps. Scale bar is 20 nm. (C) A line-scan of the EDX signal obtained from the region in the panel (B) showing the relative composition of the elements present in the film.

S3 XRD reciprocal mapping

A reciprocal space map (RSM) was taken on the X'Pert³ MRD around the symmetric $CrTe_2$ (002) peak using a PIXcel 3D detector in static line mode (shown in Fig. S3). The resulting RSM shows little mosaic disorder, consistent with the measured rocking curve width. Attempts to measure an RSM on an asymmetric peak were unsuccessful because the film was so thin; thus, the amount of strain could not be determined.

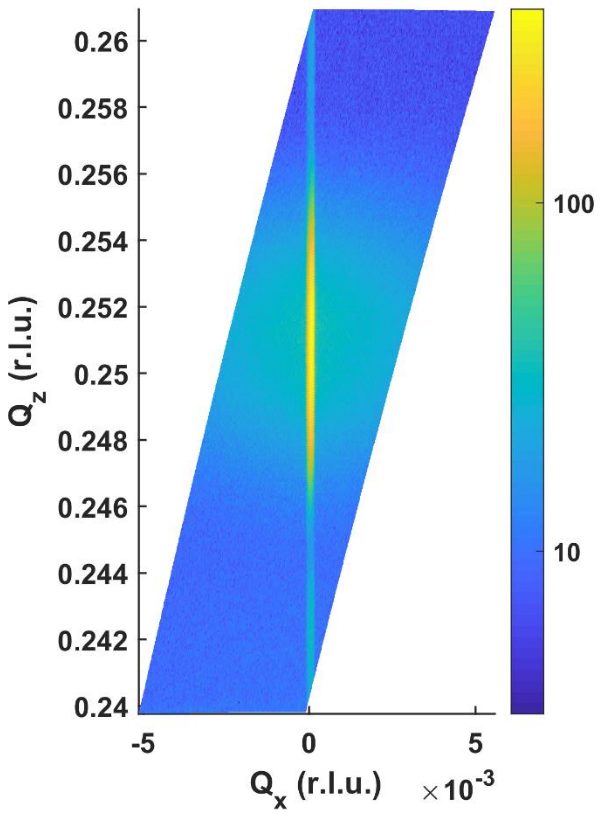

Figure S3. Reciprocal space map of the $CrTe_2$ (002) peak of a sapphire/$CrTe_2$(12u.c.)/Te sample.

S4 Comparison between the ARPES results and the DFT calculation

The comparison of the ARPES spectrum for the 12u.c. $CrTe_2$ thin film makes use of the computed Fermi level from PBE-based density functional theory including spin-orbit coupling and finite Hubbard U, using the computed band structure of the bulk plotted along the Γ-M-K plane. Here we consider effects related to k_z dispersion and also discuss the possibility of charge transfer or extrinsic doping shifting the Fermi level. Figure S4A and S4B shows the band structure of bulk 1T-$CrTe_2$ calculated by DFT, with Figure S4B providing Brillouin zone sampling that also extends in the *z* direction (Γ to A). This k_z dispersion should lift slightly the band at Γ around –1.2 eV and also drop slightly the hole-like bands around the Fermi level; both these changes would correct small discrepancies in the comparison of theory and experiment. While bulk calculations can give a sense of the effects of *z*-axis dispersion, an explicit multilayer calculation for multilayer $CrTe_2$ can provide a more direct sense of these effects. Figures S4C, D and E show the bands of a 3 u.c. $CrTe_2$ slab, color-coded so that bands that project onto the top and bottom layers are red and blue (combining to purple) while those projecting onto the middle layer are green. We focus on the purple (i.e. near-surface) bands, and show three possible choices of Fermi level corresponding to shifts of 0, 0.75 and 1 eV. Both non-zero shifts place one or more purple bands into the signal-free region around half a volt below the experimental Fermi level; these discrepancies suggest that the unshifted Fermi level presented in the main text is the most likely band alignment.

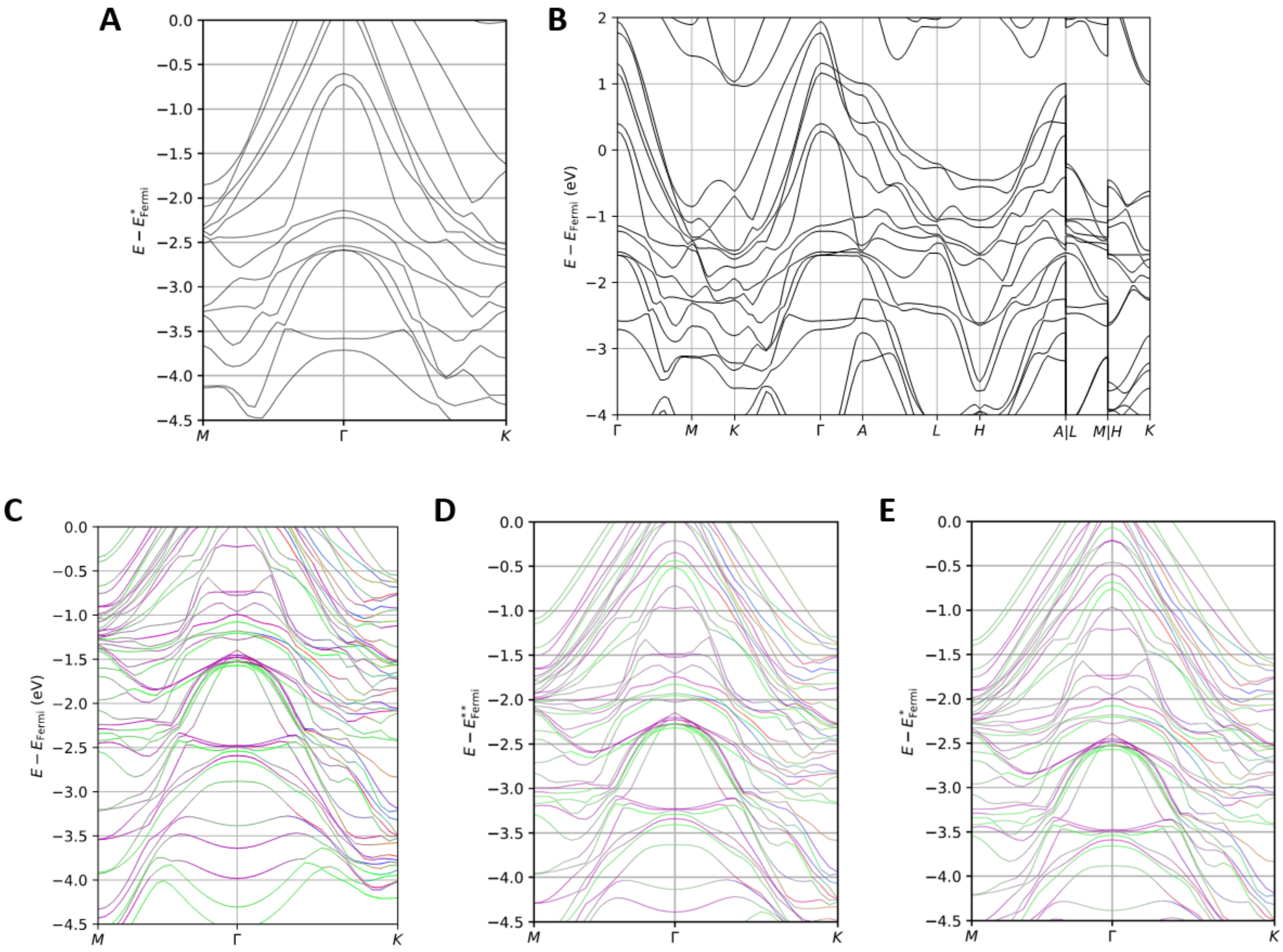

Figure S4. (A and B) DFT calculated band structures for bulk $CrTe_2$. (C-E) DFT results for 3 u.c. of $CrTe_2$ with different energy windows. The color coding comes from the projection to different layers. Red for the 1st layer, blue for the 2nd and green for the 3rd. Purple is the combination of red and blue. The M and K points are 0.92 and 1.06 Å^{-1} respectively. $E^{*}_{\text{Fermi}} = E_{\text{Fermi}} + 1$, $E^{**}_{\text{Fermi}} = E_{\text{Fermi}} + 0.75$.

S5 DFT calculations of the magnetic anisotropy energy

To understand the out-of-plane magnetic anisotropy in our $CrTe_2$ thin films, we calculated the magnetic anisotropy energy (MAE) for $CrTe_2$ as a function of the in-plane lattice constant (*a*) with different electron doping levels in Fig.S5. The MAE is demonstrated in the form of E_{in}-E_{out} where E_{in} could either be the energy for spins lying in the x or y-directions. The positive value in Fig. S5 indicates an out-of-plane easy axis. Our calculated results indicate that the energy difference between out-of-plane and in-plane directions is much larger than that between the two in-plane directions, *x* and *y*, in good agreement with previous works [65,66]. While the observed out-of-plane anisotropy in our $CrTe_2$ samples is consistent with existing experimental works [32,34], we notice that MAE can be quite sensitive to the lattice constant *a*, electron doping level and the choice of Hubbard U [34], which suggests that the easy axis direction is hard to be determined from the theoretical point of view due to its sensitivity to the experimental parameters.

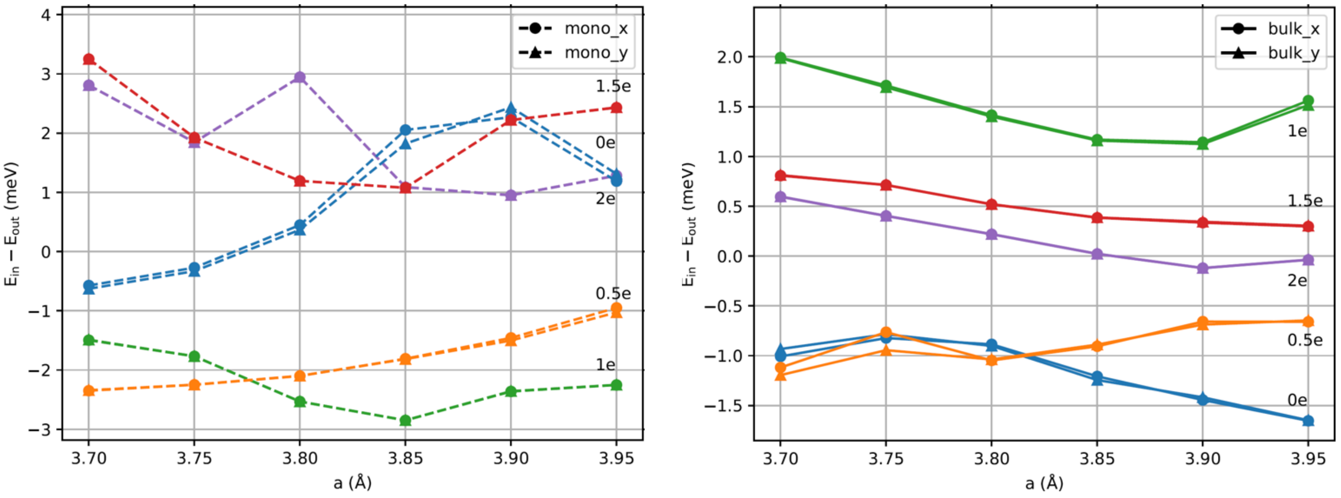

Figure S5. MAE as a function of lattice constant *a* with different electron doping levels. E_{out} is the energy for spins along out-of-plane (z) direction, and E_{in} is for the in-plane (x/y) direction.

“mono”/“bulk” refers to monolayer/bulk calculations. The lattice constant *c* is fixed at 15 Å for monolayer and 6.02 Å for bulk calculations.

S6 Longitudinal resistivity measurements of the 1T-$CrTe_2$ epilayer

Figure S6 shows the temperature dependent longitudinal resistivity of a sapphire/$CrTe_2$(12u.c.)/Te sample measured in a PPMS apparatus in a four-terminal configuration on a 1 mm x 0.5 mm Hall bar. At room temperature (300K), the resistivity of the $CrTe_2$ is $\rho_{xx} \approx 594\ \mu\Omega$.cm.

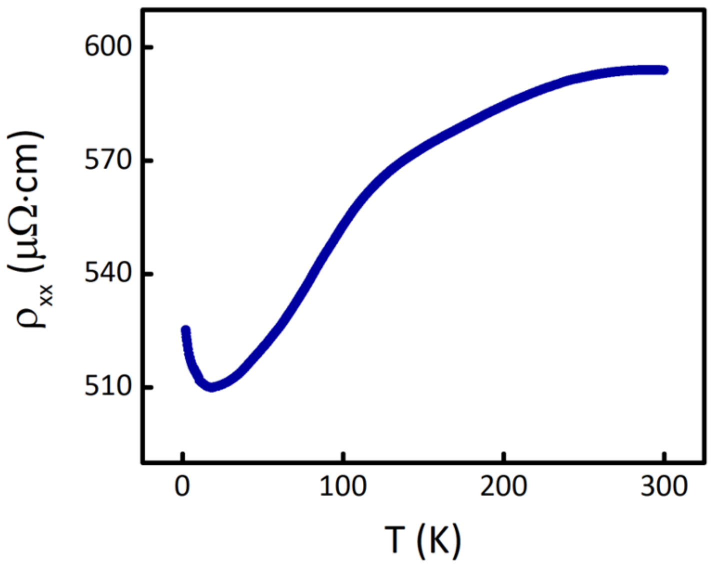

Figure S6. Temperature dependent longitudinal resistivity of a sapphire/$CrTe_2$(12u.c.)/Te sample.

S7 Attempting fits to the THE signal by using the two AHE model and field-angle dependence measurements

As we showed in Fig. 3 in the main text, the AHE measured in both 1T-$CrTe_2$ layer and $ZrTe_2$/$CrTe_2$ heterostructures can show behavior consistent with a THE arising from Berry curvature in real space. However, such behavior can also arise from coexisting anomalous Hall signals with opposite signs. Such an interpretation has been used to explain an unconventional THE-like anomalous Hall resistance in magnetically-doped topological insulator thin films and in oxide interfaces [68,69]. To test this scenario, we carried out a similar fitting process to our THE signal by assuming two AH effects following Ref.[46,68]. For this purpose, the signals were fitted to:

$$R_{yx}(H) = R_{AH1}\tanh\left(\frac{H \pm H_{c1}}{H_{01}}\right) + R_{AH2}\tanh\left(\frac{H \pm H_{c2}}{H_{02}}\right) .$$

Here, R_{AH1} (R_{AH2}) is the first (second) anomalous Hall resistance contribution with H_{c1} (H_{c2}) being the coercivity and H_{01} (H_{02}) being a scaling factor, respectively. Examples of the best fits compared to the original data are plotted in Fig. S7A, where we see that the fits do not reproduce all the details of the original data. Fig. S7B summarizes the fitted parameters as a function of temperature up to where the THE signal disappeared in our transport measurement. The variation of the fitting parameters with temperature does not yield a physically meaningful picture: for example, while R_{AH2} increases with decreasing temperature, R_{AH1} however *decreases* with decreasing temperature even though its corresponding coercive field H_{c1} and scaling factor H_{01} do not decrease. The scaling factor H_{02} also shows as a non-monotonic behavior. This suggests that the competing AHE picture is probably not a valid one.

We also performed a field-angle dependent measurement to determine the influence of the magnetic field's direction on the THE signal. Figure S7C shows that the THE signal is relatively

insensitive to the field angle up to around 45° and then disappears at higher field angles. The field-angle behavior of the THE effect is similar to what is observed in Ref.[46] and it may be related to the energy rebalancing process as the magnetic field is rotated. As we mention in the main text, direct magnetic imaging such as low temperature MFM and Lorentz TEM measurements will be needed to further determine the nature of the THE signal observed in our 1T-$CrTe_2$ thin films.

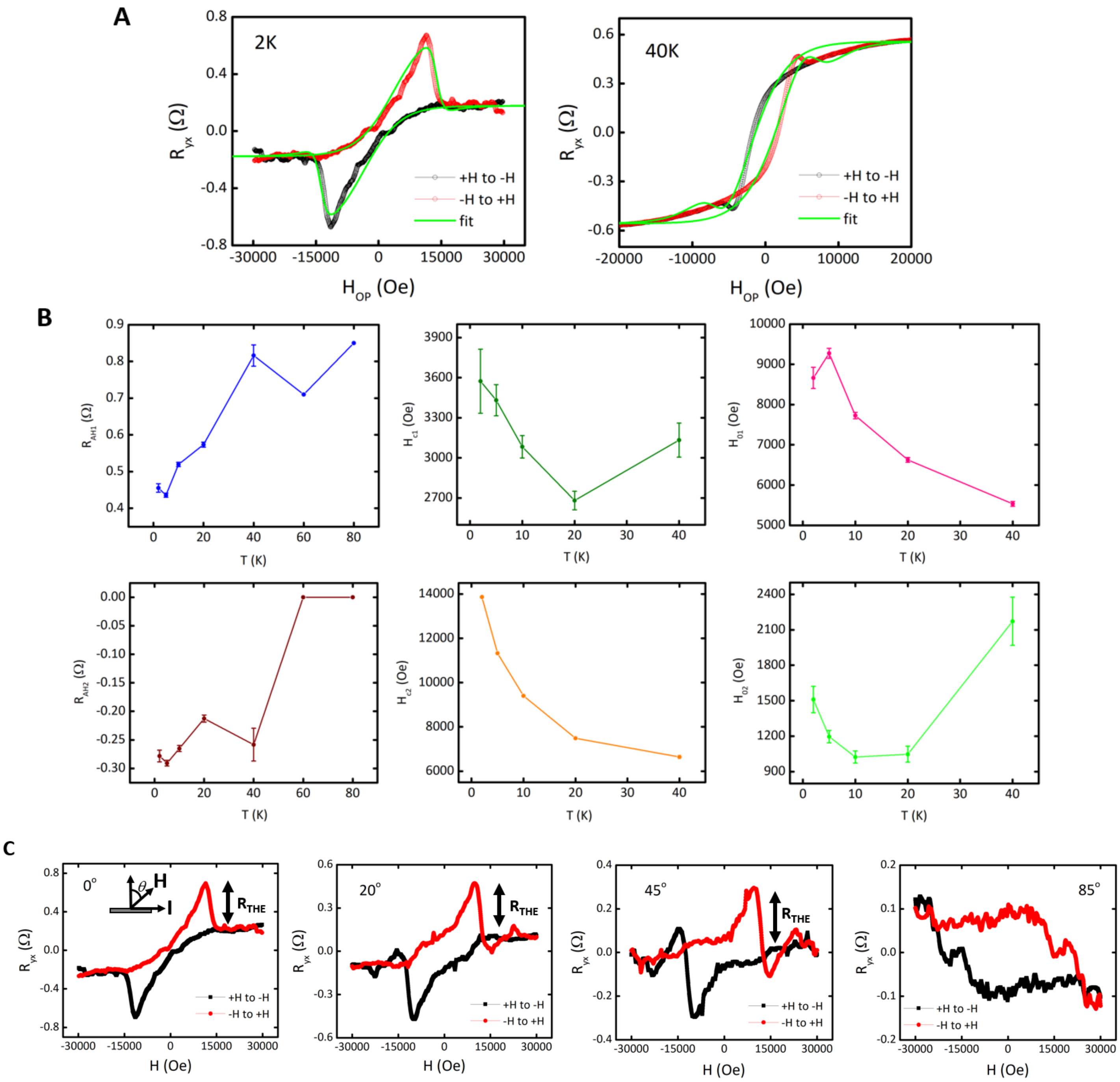

Figure S7. (A) Fits to the Hall signals of the sapphire/$CrTe_2$(12u.c.) sample using the bi-AHE models. (B) Temperature dependent fitting parameters attained from the bi-AHE fits. The error bars represent the standard errors from the best fit at each temperature as shown in (A). (C) Field-angle dependence of the Hall signals of the sapphire/$CrTe_2$(12u.c.) sample at 2K.

S8. Longitudinal magnetoresistance and magnetometry measurements on sapphire/$CrTe_2$ and $ZrTe_2$/$CrTe_2$

Figure S8A to D shows the longitudinal magnetoresistance measurements on the four samples presented in the main text within the temperature ranges where AHE is observed. Note that the higher noise level in ultrathin $CrTe_2$ sample grown on $ZrTe_2$ is likely due to the shunting effect from $ZrTe_2$. In figure S8E, we present an example of the magnetometry measurement on one sample, sapphire/$CrTe_2$(12u.c.), using a vibrating-sample-magnetometer-SQUID with in-plane-magnetic-field configuration. We were unable to carry out measurements with field along the easy axis (normal to the sample plane) because it is difficult to dice the samples grown on sapphire substrates to the requisite size for mounting in the VSM-SQUID magnetometer. Caution has been taken during the whole measurement process by avoiding any metallic contact to the sample to minimize contamination, including from unloading the sample from MBE system, sample transfer and sample preparation and mounting on the VSM setup. A value of magnetization in this $CrTe_2$ sample at T = 10 K, M_s=0.1x10^6 A/m, can be estimated. However, we cautiously point out the accuracy of such kind of magnetometry measurements is likely concerning since we observed that there could be a substantial, sample-dependent non-linear background signal when performing the $CrTe_2$ VSM, an issue even more prominent for ultrathin $CrTe_2$ samples due to their low total magnetic moment. Therefore, while the example here (Fig. 8E) might serve as a measure of the magnetization of ultrathin $CrTe_2$ films, we would like to emphasize that the AHE presented in the main text is a more reliable measure of the magnetic order in our ultrathin $CrTe_2$ thin films.

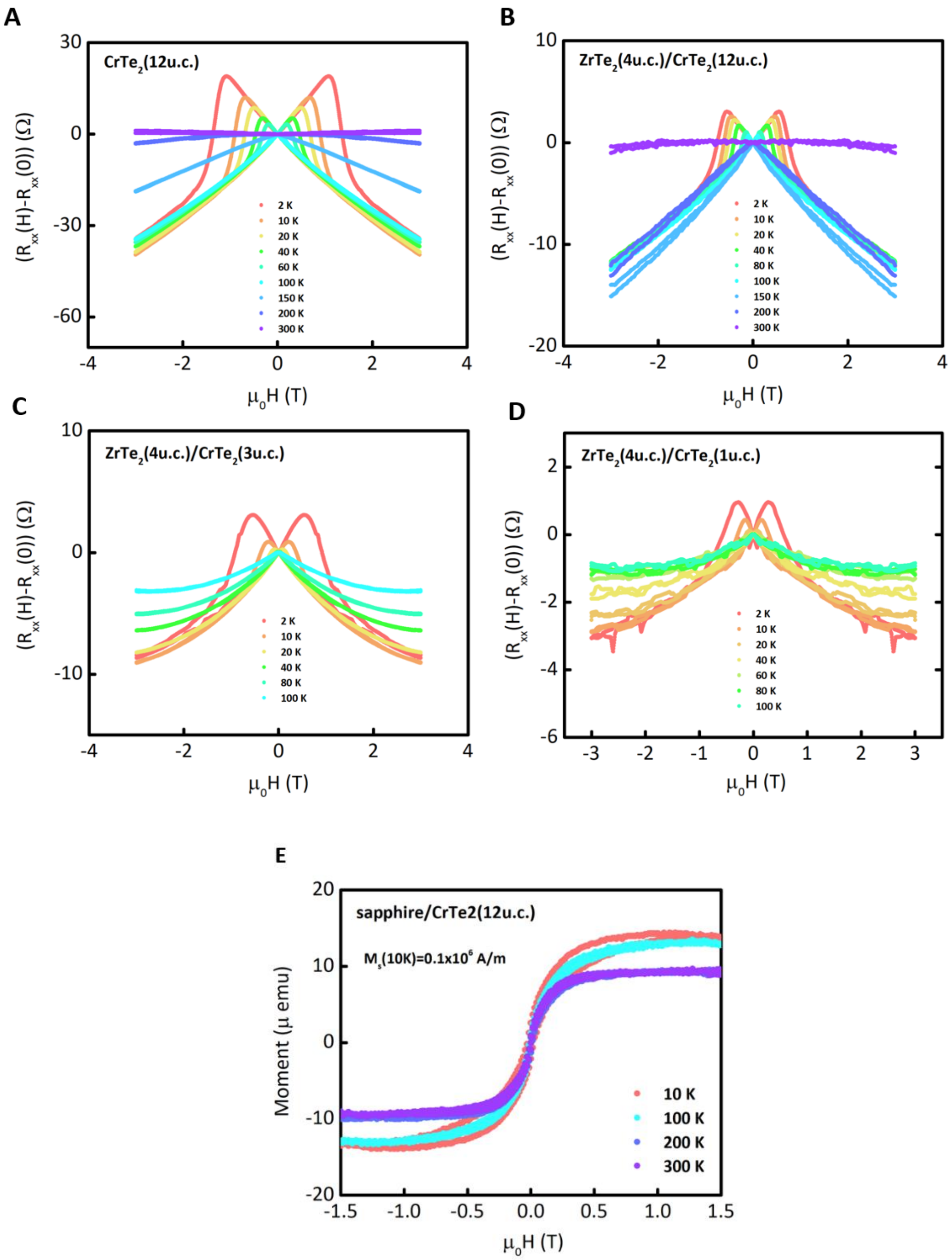

Figure S8. (A)-(D) The longitudinal magnetoresistance measurements on sapphire/$CrTe_2$(12u.c.), $ZrTe_2$(4u.c.)/$CrTe_2$(12u.c.), $ZrTe_2$(4u.c.)/$CrTe_2$(3u.c.), $ZrTe_2$(4u.c.)/$CrTe_2$(1u.c.), respectively. (B) Magnetometry measurements on a sample of sapphire/$CrTe_2$(12u.c.). The magnetic field is in plane giving a hard axis magnetization response.

S9. Current-induced magnetization switching in $ZrTe_2/CrTe_2$

By measuring the anomalous Hall resistance to determine the magnetization state of $CrTe_2$, we tested the current-induced magnetization switching of the $ZrTe_2$(8u.c.)/$CrTe_2$(3u.c.) devices between 10K and 90K under various external in-plane magnetic field along the electrical current direction, as shown in Fig. S9. The step-like switching edges during most of these switching attempts are consistent with the domain nucleation and domain wall motion, probably due to the micro-scale size of the devices.

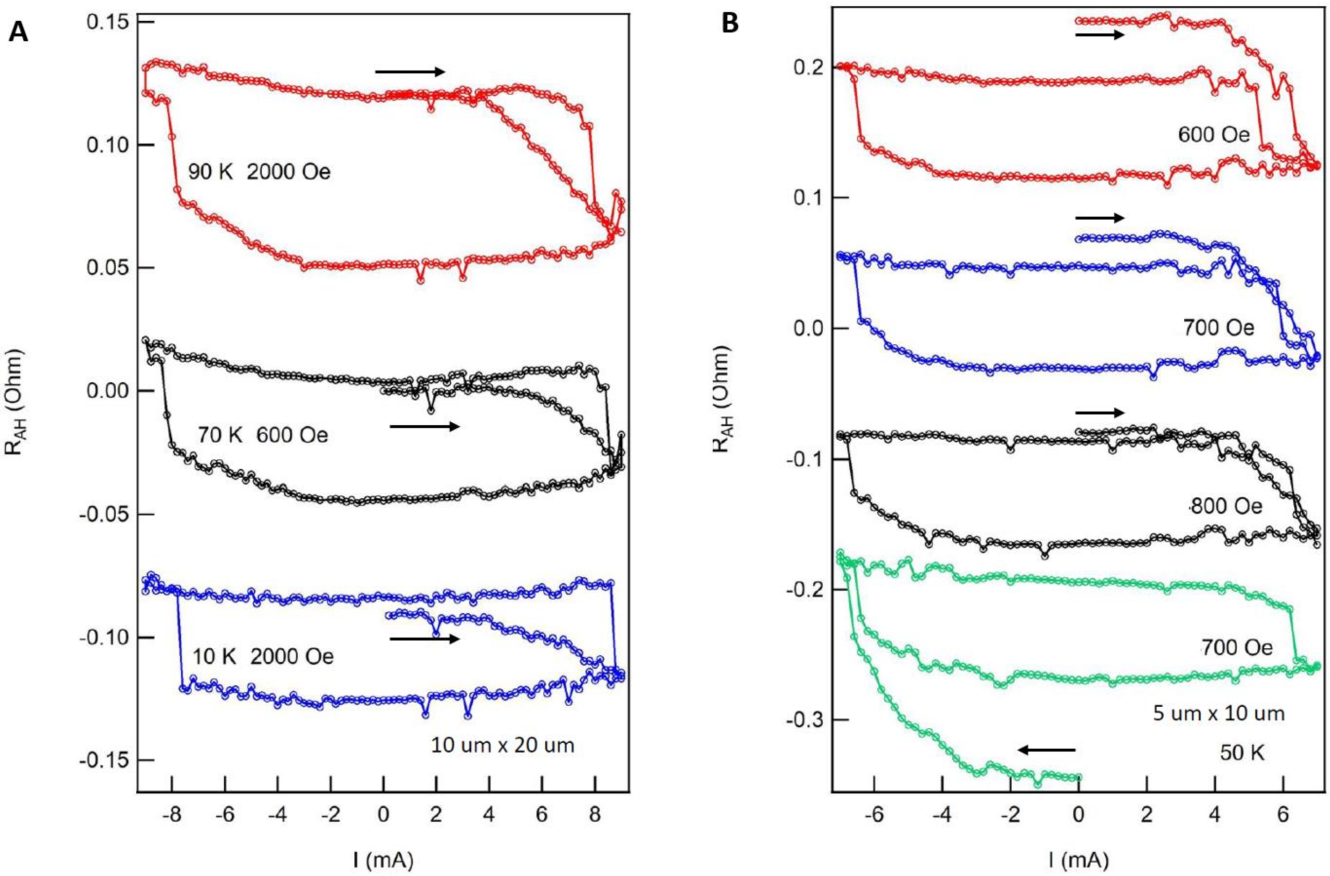

Figure S9. (A) Current-induced magnetization switching measurements at 10K, 70K and 90K. (B) Current-induced magnetization switching measurements at 50K under various in-plane magnetic field. The arrows indicate the starting positions.

S10 DFT calculations of SHC of $ZrTe_2$ thin films

Figure S10 shows the atomic structure, band structure, and energy dependent SHC of monolayer and bilayer $ZrTe_2$ thin films. The band structure of monolayer and bilayer systems is similar to that of the trilayer system. The Fermi level in thin films is clearly shifted to higher energy compared to the bulk phase, which is caused by the change of band dispersion especially along the out-of-plane direction due to the quantum confinement effect.

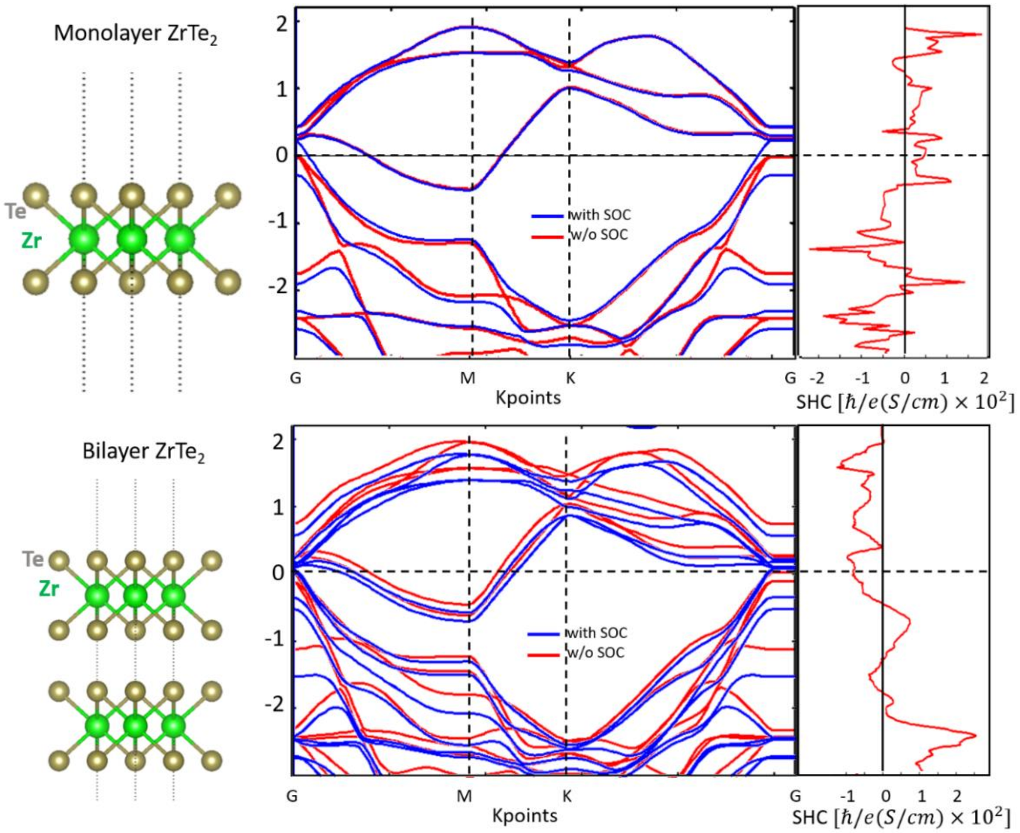

Figure S10. DFT calculated band structure and spin Hall conductivity of the monolayer (upper panel) and bilayer (lower panel) $ZrTe_2$ thin films, respectively. Blue and red lines corresponding to band structure with and without spin-orbit coupling, respectively.
